# Attention Deficit Hyperactivity Disorder Symptoms and Low Educational Achievement: Evidence Supporting A Causal Hypothesis

**DOI:** 10.1007/s10519-017-9836-4

**Published:** 2017-02-13

**Authors:** Eveline L. de Zeeuw, Catharina E. M. van Beijsterveldt, Erik A. Ehli, Eco J. C. de Geus, Dorret I. Boomsma

**Affiliations:** 1 Department of Biological Psychology, Vrije Universiteit Amsterdam, Van der Boechorststraat 1, 1081 BT Amsterdam, The Netherlands; 2 Amsterdam Public Health research institute, VU Medical Centre, Amsterdam, The Netherlands; 3 Avera Institute for Human Genetics, Sioux Falls, SD USA

**Keywords:** ADHD, Educational achievement, Causality, Genetic pleiotropy, Twin study, Methylphenidate

## Abstract

Attention Deficit Hyperactivity Disorder (ADHD) and educational achievement are negatively associated in children. Here we test the hypothesis that there is a direct causal effect of ADHD on educational achievement. The causal effect is tested in a genetically sensitive design to exclude the possibility of confounding by a third factor (e.g. genetic pleiotropy) and by comparing educational achievement and secondary school career in children with ADHD who take or do not take methylphenidate. Data on ADHD symptoms, educational achievement and methylphenidate usage were available in a primary school sample of ~10,000 12-year-old twins from the Netherlands Twin Register. A substantial group also had longitudinal data at ages 7–12 years. ADHD symptoms were cross-sectionally and longitudinally, associated with lower educational achievement at age 12. More ADHD symptoms predicted a lower-level future secondary school career at age 14–16. In both the cross-sectional and longitudinal analyses, testing the direct causal effect of ADHD on educational achievement, while controlling for genetic and environmental factors, revealed an association between ADHD symptoms and educational achievement independent of genetic and environmental pleiotropy. These findings were confirmed in MZ twin intra-pair differences models, twins with more ADHD symptoms scored lower on educational achievement than their co-twins. Furthermore, children with ADHD medication, scored significantly higher on the educational achievement test than children with ADHD who did not use medication. Taken together, the results are consistent with a direct causal effect of ADHD on educational achievement.

**Electronic supplementary material:**

The online version of this article (doi:10.1007/s10519-017-9836-4) contains supplementary material, which is available to authorized users.

## Introduction

With a prevalence rate of 3–7% in school-aged children, Attention Deficit Hyperactivity Disorder (ADHD) is relatively common in childhood (American Psychiatric Association 2000; Thomas et al. 2015). ADHD is characterized by problems with attention in combination with hyperactivity and/or impulsivity. A negative relationship between ADHD and educational achievement has been observed in both clinical and population samples (Polderman et al. 2010).

Genetic studies have established that ADHD is amongst the most heritable psychiatric childhood disorders (Hudziak et al. 2007; Lubke et al. 2009) with estimates of heritability in children of over 70% (Burt 2009; Derks et al. 2009). The extent to which genetic factors contribute to the variation in inattention and hyperactivity is comparable (Nikolas and Burt 2010). There is no evidence that environmental factors shared between children growing up in the same family have a significant influence (Burt 2009). Educational achievement is characterized by a moderate to high heritability with a small influence of shared environmental factors (Bartels et al. 2002; de Zeeuw et al. 2015; Haworth et al. 2011).

Bivariate twin studies have estimated significant genetic correlations between the number of ADHD symptoms and educational achievement (Greven et al. 2014; Saudino and Plomin 2007). A genetic correlation between ADHD and educational achievement was confirmed by a significant prediction of ADHD symptoms in children by polygenic scores based on the effect sizes of genetic variants related to educational attainment in adults (de Zeeuw et al. 2014). Polygenic scores for clinical ADHD, in turn, predicted intelligence, which is one of the strongest predictors of educational achievement (Martin et al. 2014).

There are at least three explanations for a genetic correlation between ADHD symptoms and educational achievement. One explanation is that there is a direct causal effect of ADHD on educational achievement. The genetic variants influencing ADHD would then, indirectly through the causal chain, also have an effect on educational achievement. The second explanation is reverse direct causality where bad performance at school enhances ADHD symptoms. A third explanation is that some other underlying factor influences both ADHD and educational achievement. Genetic pleiotropy, which occurs when the same genes, through the same underlying biological mechanisms, affect both phenotypes, is a potential candidate. A direct causal effect, a reverse direct causal effect and genetic pleiotropy are not mutually exclusive and could all be present.

In the present study a direct causal effect of ADHD on educational achievement is tested taking potential genetic confounding into account in a large population-based sample of children in primary school. What must be noted is that a direct causal effect can only be refuted and not proven. There are several predictions that follow from a direct causal effect.

**Fig. 1:**
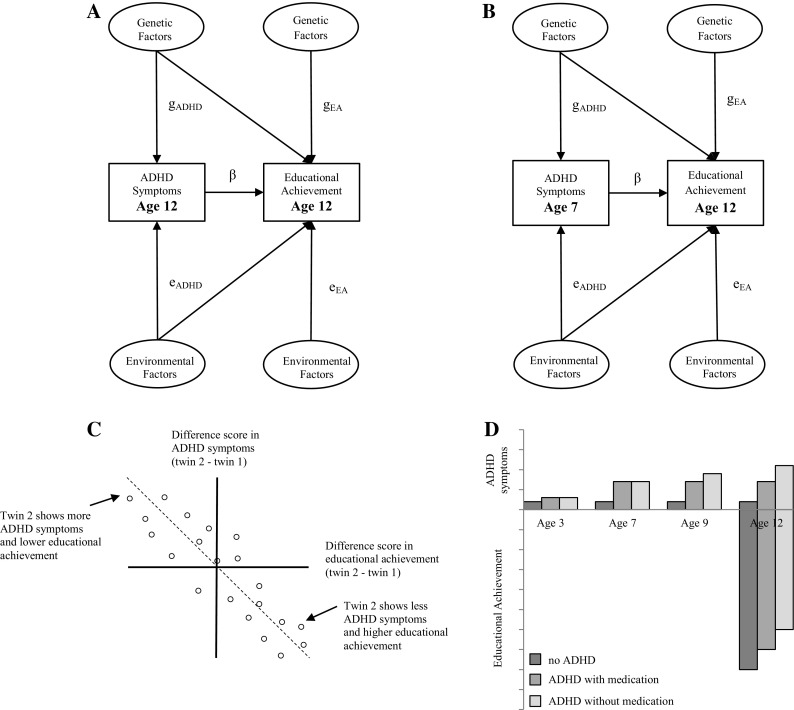
Graphical representation and expected results of the models testing the causal effect of ADHD symptoms on educational achievement. Cross-sectional bivariate twin and within-person regression model (**a**), longitudinal bivariate twin and within-person regression model (**b**), MZ twin intra-pair differences model (**c**) and between groups (i.e. children without ADHD, children with ADHD using medication, children with ADHD not using medication) differences model (**d**)

A first prediction is that if ADHD symptoms have a direct causal effect on educational achievement, ADHD symptoms will have an effect on educational achievement even when taking into account genetic and environmental factors with a pleiotropic influence on ADHD symptoms and educational achievement. A direct path between two phenotypes and the correlations between the latent genetic and environmental factors influencing the two phenotypes can be estimated with a bivariate twin and within- person regression model (Kohler et al. 2011; Turkheimer and Harden 2014) (Fig. 1a). This provides the possibility to estimate a direct causal effect of ADHD symptoms on educational achievement in the presence of genetic pleiotropy and environmental correlations by including a within-person regression of educational achievement at age 12 on ADHD symptoms at age 12. A non-significant direct path from ADHD symptoms to educational achievement would fail to support the hypothesis of a direct causal effect. If the direct path is not significant and only the genetic correlation is significant this would point to genetic pleiotropy.

A second prediction is that the association between ADHD symptoms and educational achievement exists longitudinally and that there is a direct path from ADHD symptoms at an earlier age to educational achievement at a later age. The prediction based on this hypothesis can be tested in the previously explained bivariate twin model that includes a within-person regression of educational achievement at age 12 on ADHD symptoms at age 7 (Kohler et al. 2011; Turkheimer and Harden 2014) (Fig. 1b).

A third prediction is that, within monozygotic (MZ) twin pairs, differences in ADHD symptoms should be associated with differences in educational achievement because confounding by genes and a shared environment is excluded. Twins from MZ pairs, who show more ADHD symptoms than their co-twins should perform worse in school. A non-significant association would indicate that genetic or environmental factors are driving the association (De Moor et al. 2008) (Fig. 1c).

A fourth prediction is that lowering of the number of ADHD symptoms will improve educational achievement. Methylphenidate, which is a stimulant drug that increases the levels of the neurotransmitter dopamine in the brain, has been shown to lead to a reduction in symptoms of ADHD in the majority of children (Biederman et al. 2004; King et al. 2006; Schachter et al. 2001). However, evidence for long-term benefits of methylphenidate with regard to educational achievement is inconsistent (Prasad et al. 2013; Sharp 2014). A significantly higher educational achievement test score in children with ADHD who use methylphenidate than children with ADHD who never used methylphenidate would be consistent with the causal hypothesis. The hypothetical pattern of results that is expected under the direct causal hypothesis of ADHD on educational achievement is displayed in Fig. 1d.

## Methods

### Participants

The Netherlands Twin Register (NTR), established around 1987 by the department of Biological Psychology at the VU University Amsterdam, registers approximately 40% of all multiple births in the Netherlands (Bartels et al. 2007; van Beijsterveldt et al. 2013). The NTR collects longitudinal data by surveys at specific ages. Parents of twins receive surveys about the development of their children every 2 years until the twins are 12 years old. Parents are also asked consent, at ages 7, 9, and 12, to contact the primary school teacher(s) of their children. After parents give their permission, the children themselves are also invited to fill out a survey at the ages 14 and 16.

Children with a disease or handicap that interfered severely with daily functioning were excluded from this study. The total sample included children with data on educational achievement at age 12 and on ADHD symptoms at age 7 and 12 (N = 3868), children with data on educational achievement and on ADHD symptoms at one of the ages (12: N = 4919; 7: N = 671), children with only data on educational achievement (N = 885) and children with only data on ADHD symptoms (7 and 12: N = 1786; 12: N = 2616; 7: N = 6799). The total sample included 3549 twin pairs of opposite sex. For the same-sex twin pairs, determination of zygosity status was based on DNA polymorphisms (N = 1244) or on the basis of parental report of items on resemblance in appearance (N = 6150). The parental report establishes zygosity with an accuracy of 93% (Rietveld et al. 2000).

To test the effect of methylphenidate on ADHD symptoms and educational achievement we identified three groups in the cross-sectional sample of 12-year-olds, i.e. a group of children with ADHD prescribed methylphenidate, a group of children with ADHD not on methylphenidate and a control group of children without ADHD. ADHD status was inferred from medication usage or a high score on the ADHD index of the Conners’ Parental Rating Scale. Children taking methylphenidate at age 12 were classified as the ADHD group with medication (N = 349). From the group of children that never took methylphenidate, children with a T-score above 70 (‘atypical’ range) were classified as the ADHD group without medication (N = 534) and the children with a T-score below 56 (‘normal’ range) were classified as controls (N = 10,019). T-scores of the ADHD index were based on the total sample and were determined separately for boys and girls (Conners 1997; Kumar and Steer 2003).

### Measures

ADHD symptoms were assessed, by mothers and teachers, with the short version of the Conners’ Rating Scales - Revised (CRS-R). The CRS-R included scales measuring Inattention (ATT - parent: 6 items; teacher: 5 items), Hyperactivity (HYP - parent: 6 items; teacher: 7 items) and ADHD index (ADHD - parent: 12 items; teacher: 12 items) (Conners 2001). The Cronbach’s alpha of the ADHD index scale was 0.91 and the scale has been shown to be able to discriminate between children with an ADHD diagnosis and children without ADHD in a clinical population (Kumar and Steer 2003). Data collection of the CRS-R only started in 2002, resulting in ADHD symptoms at age 7 being available for cohorts 1994–2005 and ADHD symptoms at age 12 for cohorts 1989–2000. Sum scores were computed when there were a limited number of missing items for a scale. Missing items were imputed by the averaged item score of the scale for that child. The sum scores of the scales showed an L-shaped distribution and the data were square root transformed and standardized prior to the bivariate twin and within-person regression analyses (Derks et al. 2004).

ADHD symptoms were also rated by mothers with the Attention Problems scale (1½–5 years: 5 items; 6–18 years: 10 items) of the Child Behavior Check List (CBCL). These data were available from age 3 onwards, when the children did not yet attend any form of education (Achenbach 1991).

Data on stimulant medication usage, methylphenidate, came from two distinct sources, i.e. parental reports and records of insurance claims of one of the large insurance company’s, which insures approximately 25% of all people in the Netherlands (Ariel et al. 2015). The sources were to a large degree in agreement with each other. None of the mothers reported that a child was using methylphenidate without an actual claim being registered in the insurance records. In some instances, the records of the insurance company reflected that ADHD medication was prescribed while the mother did not report the methylphenidate usage (~20%). However, this is not necessarily a contradiction since a child might have been prescribed another type of ADHD medication or might not actually be taking the prescribed medication.

Educational achievement was assessed by a nationwide educational achievement test (Cito 2002). The test consists of multiple choice items in four domains, namely Arithmetic, Language, Study Skills and Science and Social Studies (optional). Performance in the different domains was measured with the percentage of correctly answered items. The first three test scales are combined into a Total Score, which is standardized on a scale between 500–550. The data were standardized prior to the bivariate twin and within-person regression analyses. The internal reliability of the test is good (*α*=0.95) as is the test–retest reliability (*r* = 0.96) (van Boxtel et al. 2011). Finally, the self-reports of the level of secondary education that children attended at the age of 14 and/or 16 was evaluated. In the Netherlands, there are three levels of secondary education, vocational education (level 1), general higher education (level 2) and preparatory academic education (level 3) (Cito 2002).

Parental socioeconomic status (SES) was based on a full description of the occupations of both parents and coded according to the system from the Statistics Netherlands (CBS 2001) or by the EPG-classification scheme (Erikson et al. 2010). The classification of SES was based on the mental complexity of the work on 5 levels (ranging from a low level job to occupation on academic level).

### Statistical analyses

The hypothesis of a direct causal effect of ADHD symptoms on educational achievement was tested in four models, i.e. a cross-sectional combined bivariate twin and within-person regression model, a longitudinal combined bivariate twin and within-person regression model, a MZ twin intra-pair differences model and a between groups differences model. All analyses, except for the second, which was based on longitudinal data, were based on the cross-sectional data.

In a series of univariate saturated models, means and variances, by zygosity and gender, were estimated by maximum likelihood in the R (R Core Team 2014) package OpenMx Version 2.5.2 (Boker et al. 2011, 2012). Constrains were tested, including equality of means and variances across zygosity and gender. Next, univariate ACE and ADE models were fitted to the data. Additive genetic factors (A) represent the summation over all additive genetic effects. Dominant genetic factors (D) result from the interaction between alleles. Environmental factors consist of all non-genetic influences, including prenatal factors, and can be distinguished into common (C) and unique environmental (E) factors. Common environmental factors are influences that are shared between twins or siblings and increase their similarity. Unique environmental factors are influences, including measurement error, that are not shared between twins and make them different (Plomin et al. 2008; Posthuma et al. 2003). In subsequent models, we tested whether genetic and environmental factors influenced variation in boys and girls to the same extent by fitting a model, which incorporated total variance differences, but constrained standardized variance components to be the same across gender. The significance of C or D was tested by dropping them from the model. The difference in fit between nested models was assessed by log-likelihood ratio tests (LRT). The difference in −2log-likelihood (−2LL) between models follows a ${{\chi }^{2}}$ distribution, which is tested against the difference in degrees of freedom (df) between models. Next, a series of bivariate saturated models was fitted to the data to estimate the within-phenotype and cross-phenotype twin correlations.

A combined cross-sectional bivariate twin and within-person regression model estimated the association between ADHD symptoms, as assessed by mothers, at age 12 and educational achievement at age 12 and tested if it could be explained by a direct effect of ADHD symptoms on educational achievement or by genetic and environmental correlations (Kohler et al. 2011; Turkheimer and Harden 2014) (see Fig. 1a). Bivariate models allow the estimation of genetic and environmental correlations between two phenotypes. Under some conditions, the model also allows the identification of a direct path from one phenotype to the second phenotype. Because there is a difference in the genetic model underlying ADHD (ADE model) and educational achievement (ACE model), the full bivariate model was identified. When the direct path is significant this would be compatible with the direct causal hypothesis of ADHD symptoms on educational achievement.

In case of causality there should also be a significant longitudinal association between ADHD symptoms, as assessed by mothers, at the beginning of primary school and subsequently, a 5-year time interval, educational achievement. The genetically sensitive sample in this study can again test the direct causal hypothesis of ADHD on educational achievement over time in a longitudinal bivariate twin and within-person regression model (see Fig. 1b). The direct causal hypothesis would be supported when the direct path between ADHD symptoms at age 7 and educational achievement at age 12 is significant while just a significant genetic correlation would indicate genetic pleiotropy.

The MZ twin intra-pair differences model tested the association between differences in ADHD symptoms, as assessed by mothers, and differences in educational achievement within MZ twin pairs (Fig. 1c) by correlating the difference in ADHD symptoms within MZ pairs with the differences in educational achievement. A significant association would support the causal hypothesis while a non-significant association is more compatible with genetic pleiotropy.

The between groups differences model tested the association between ADHD symptoms and educational achievement in three groups of children, i.e. of children with ADHD who were prescribed methylphenidate, a group of children with ADHD not taking methylphenidate and a group of children without ADHD. ADHD symptoms, as rated by mothers and teachers, and educational achievement were standardized and regressed, using dummy coding, on group membership, using generalized linear models in SPSS (IBM Corp 2015). To investigate whether the groups, i.e. no-ADHD, ADHD with medication and ADHD without medication, differed in the level of secondary education and to check for differences in SES, data were analyzed by ordinal logistic generalized linear models with group membership, using dummy coding, as predictor and level of secondary education and parental SES as outcomes. To correct for dependency of the observations due to family clustering family membership was added as a random effect to all the models. A significant higher educational achievement test score in  the ADHD group with medication compared to the ADHD group without medication would provide support for a causal effect of ADHD symptoms on educational achievement. The total number of independent dimensions in the outcome data was extracted from the correlation matrix of the phenotypes with the MatSpD program (Li and Ji 2005; Nyholt 2004). The phenotypes contained 7 independent dimensions and therefore a p-value of 0.007 (0.05/7) was considered significant.

## Results

There were no mean differences in ADHD symptoms, except at age 12, between MZ and DZ twins, and no variance differences, with the exception of hyperactivity at age 12, excluding possible sibling contrast effects. There were mean gender differences with boys showing more ADHD symptoms, both inattention and hyperactivity, than girls. Boys scored higher on the educational achievement test. Variances for ADHD symptoms differed between boys and girls, but this was not the case for educational achievement (Table S1 and S2).

The twin correlations as estimated in saturated models are summarized in Table 1. All twin correlations were significant and all MZ correlations were higher than the DZ correlations. For ADHD symptoms, the MZ correlations were often more than twice as high as the DZ correlations, which suggests an influence of D, while for educational achievement the DZ correlations were larger than half the MZ correlations, indicating the presence of C.

**Table 1:** Twin correlations (95% Confidence Interval) [N] for ADHD symptoms and educational achievement (EA) Cross-sectional and longitudinal cross-twin within-phenotype and cross-twin cross-phenotype correlations

	Inattention	Hyperactivity	ADHD	Educational achievement
Cross-sectional cross-twin within-phenotype				
ADHD and EA at age 12				
MZm	0.77 (0.75; 0.80) [1065]	0.78 (0.75; 0.80) [1066]	0.82 (0.80; 0.84) [1071]	0.81 (0.78; 0.83) [805]
DZm	0.23 (0.18; 0.29) [1124]	0.29 (0.23; 0.34) [1136]	0.29 (0.23; 0.34) [1130]	0.46 (0.39; 0.51) [712]
MZf	0.70 (0.67; 0.72) [1176]	0.75 (0.73; 0.78) [1185]	0.75 (0.72; 0.77) [1181]	0.83 (0.81; 0.85) [966]
DZf	0.24 (0.18; 0.30) [989]	0.35 (0.29; 0.40) [1006]	0.33 (0.27; 0.38) [1001]	0.43 (0.37; 0.48) [755]
DOS	0.19 (0.15; 0.23) [2044]	0.29 (0.25; 0.33) [2061]	0.29 (0.25; 0.32) [2059]	0.44 (0.40; 0.48) [1541]
Cross-sectional cross-twin cross-phenotype				
ADHD and EA at age 12				
MZm	−0.37 (−0.42; −0.31) [1437]	−0.14 (−0.21; −0.08) [1440]	−0.29 (−0.35; −0.23) [1440]	
DZm	−0.07 (−0.12; −0.01) [1348]	−0.06 (−0.11; −0.00) [1347]	−0.06 (−0.12; −0.00) [1348]	
MZf	−0.32 (−0.37; −0.27) [1717]	−0.07 (−0.13; −0.00) [1720]	−0.26 (−0.31; −0.20) [1716]	
DZf	−0.03 (−0.09; 0.03) [1350]	−0.02 (−0.08; 0.04) [1350]	−0.03 (−0.09; 0.03) [1351]	
DOS	−0.11 (−0.15; −0.08) [2832]	−0.09 (−0.13; −0.05) [2838]	−0.09 (−0.13; −0.06) [2835]	
Longitudinal cross-twin within-phenotype				
ADHD at age 7 and EA at age 12				
MZm	0.74 (0.72; 0.77) [1096]	0.82 (0.80; 0.84) [1100]	0.77 (0.74; 0.79) [1099]	0.81 (0.78; 0.83) [805]
DZm	0.29 (0.24; 0.35) [1038]	0.39 (0.34; 0.43) [1037]	0.34 (0.29; 0.39) [1038]	0.46 (0.39; 0.51) [712]
MZf	0.74 (0.71; 0.76) [1253]	0.80 (0.78; 0.82) [1255]	0.76 (0.73; 0.78) [1254]	0.83 (0.81; 0.85) [966]
DZf	0.27 (0.21; 0.33) [977]	0.42 (0.37; 0.47) [979]	0.29 (0.23; 0.34) [979]	0.43 (0.37; 0.48) [755]
DOS	0.27 (0.23; 0.31) [2108]	0.38 (0.35; 0.42) [2108]	0.29 (0.25; 0.33) [2108]	0.44 (0.40; 0.48) [1541]
Longitudinal cross-twin cross-phenotype				
ADHD at age 7 and EA at age 12				
MZm	−0.31 (−0.39; −0.23) [731]	−0.17 (−0.26; −0.08) [733]	−0.27 (−0.35; −0.18) [733]	
DZm	−0.06 (−0.13; 0.02) [640]	−0.04 (−0.12; 0.04) [641]	−0.08 (−0.15; 0.00) [641]	
MZf	−0.22 (−0.30; −0.13) [867]	−0.05 (−0.14; 0.03) [867]	−0.19 (−0.27; −0.10) [867]	
DZf	−0.06 (−0.13; 0.02) [744]	−0.09 (−0.17; −0.01) [752]	−0.04 (−0.12; 0.04) [750]	
DOS	−0.13 (−0.18; −0.08) [1434]	−0.14 (−0.20; −0.09) [1443]	−0.12 (−0.17; −0.06) [1435]	

*Note* The total sample is used in both the cross-sectional and longitudinal analyses

*MZm* monozygotic boys, *DZm* dizygotic boys, *MZf* monozygotic girls, *DZf* dizygotic girls, *DOS* dizygotic of opposite-sex

Fitting of the alternative univariate models, ACE and ADE, confirmed that D was important for ADHD symptoms, while C had an effect on educational achievement. Some gender differences in the genetic models were observed, D had a larger effect on ADHD symptoms in girls at age 7 and in boys at age 12. Heritability of educational achievement did not differ between boys and girls. The percentages of the variance in educational achievement explained by the different variance components are reported in Table S3.

### Bivariate twin and within-person regression model

#### Cross-sectional

The cross-sectional phenotypic correlations between ADHD symptoms (CRS-R) and educational achievement are given in Table 2. All correlations were negative and between −0.16 and −0.39 for boys and −0.12 and −0.41 for girls. The magnitude of the phenotypic correlations depended on the subtype of symptoms, with larger correlations for inattention than hyperactivity. Approximately 15% of the variance in ADHD symptoms and educational achievement overlapped.

**Table 2:** Correlations (95% Confidence Intervals) [N] between ADHD symptoms and educational achievement (EA) Cross-sectional, longitudinal and MZ twin intra-pair differences correlations

	Inattention		Hyperactivity		ADHD	
Cross-sectional						
ADHD and EA at age 12						
Boys	−0.39 (−0.41; −0.36)	[4214]	−0.16 (−0.20; −0.13)	[4216]	−0.32 (−0.35; −0.29)	[4218]
Girls	−0.41 (−0.43; −0.38)	[4558]	−0.12 (−0.15; −0.08)	[4561]	−0.33 (−0.36; −0.30)	[4566]
Longitudinal						
ADHD at age 7 and EA at age 12						
Boys	−0.31 (−0.36; −0.27)	[2143]	−0.16 (−0.21; −0.12)	[2155]	−0.27 (−0.32; −0.23)	[2146]
Girls	−0.33 (−0.36; −0.28)	[2359]	−0.17 (−0.21; −0.12)	[2374]	−0.28 (−0.32; −0.24)	[2368]
MZ twin intra-pair differences						
ADHD and EA at age 12						
Boys	−0.29 (−0.35; −0.22)	[686]	−0.10 (−0.18; −0.03)	[687]	−0.32 (−0.38; −0.25)	[687]
Girls	−0.35 (−0.40; −0.28)	[835]	−0.11 (−0.18; −0.04)	[838]	−0.32 (−0.38; −0.26)	[836]

Table 3 includes the point estimates and 95% confidence intervals of the cross-sectional genetic and unique environmental correlations and the direct paths between ADHD symptoms at age 12 and educational achievement at age 12. The direct path between ADHD symptoms and educational achievement was significant ($\beta$ = −0.50, ${{\chi }^{2}}$ (1) = 32.99, *p* < 0.001). For the inattention subtype the direct path was also significant ($\beta$ = −0.42, ${{\chi }^{2}}$ (1) = 76.69, *p* < 0.001), but this was not the case for the hyperactivity subtype of symptoms ($\beta$ = 0.06, ${{\chi }^{2}}$ (1) = 0.04, *p* = 0.833). Thus, while correcting for possible confounding by genetic and environmental effects, a direct path between ADHD symptoms and educational achievement was present.

**Table 3:** Genetic and unique environmental correlations and direct paths (95% Confidence Interval) between ADHD symptoms and educational achievement (EA)

	Inattention	Hyperactivity	ADHD
Cross-sectional			
ADHD and EA at age 12			
Genetic			
Boys	0.01 (−0.08; 0.24)	−0.17 (−0.26; 0.13)	0.16 (0.01; 0.26)
Girls	−0.06 (−0.13; 0.11)	−0.12 (−0.18; 0.08)	0.07 (−0.05; 0.17)
Unique environmental			
Boys	0.07 (0.03; 0.21)	−0.06 (−0.14; 0.10)	0.07 (−0.00; 0.16)
Girls	0.05 (0.01; 0.16)	−0.05 (−0.10; 0.07)	0.07 (−0.00; 0.14)
Direct path			
Boys and girls	−0.42 (−0.67; −0.34)	0.06 (−0.31; 0.18)	−0.50 (−0.66; −0.32)
Longitudinal			
ADHD at age 7 and EA at age 12			
Genetic			
Boys	0.13 (−0.13; 0.22)	0.03 (−0.27; 0.24)	0.09 (−0.18; 0.22)
Girls	0.05 (−0.15; 0.13)	−0.01 (−0.23; 0.15)	0.02 (−0.20; 0.14)
Unique environmental			
Boys	0.16 (0.02; 0.22)	0.05 (−0.11; 0.16)	0.09 (−0.06; 0.18)
Girls	0.13 (0.01; 0.17)	0.05 (−0.09; 0.13)	0.10 (−0.04; 0.16)
Direct path			
Boys and girls	−0.51 (−0.62; −0.20)	−0.21 (−0.49; 0.21)	−0.42 (−0.58; −0.04)

#### Longitudinal

The longitudinal phenotypic correlations between ADHD symptoms (CRS-R) and educational achievement were also significant, ranging from −0.16 to −0.31 for boys and −0.17 to −0.33 for girls, and were rather comparable to the cross-sectional correlations. This means that ADHD symptoms, measured 5 years earlier, are also associated with educational achievement (Table 2).

The longitudinal genetic and unique environmental correlations and direct paths, with their 95% confidence intervals, between ADHD symptoms at age 7 and educational achievement at age 12 are displayed in Table 3. The direct path between ADHD symptoms and educational achievement was marginally significant different from zero ($\beta$ = −0.42, ${{\chi }^{2}}$ (1) = 4.47, *p* = 0.034). The direct path between the inattentive symptoms and educational achievement was significant ($\beta$ = −0.51, ${{\chi }^{2}}$ (1) = 13.62, *p* < 0.001), but this was not true for the hyperactive symptoms ($\beta$ = −0.21, ${{\chi }^{2}}$ (1) = 0.08, *p* = 0.775).

### MZ twin intra-pair differences model

The associations between MZ twin intra-pair differences in ADHD symptoms and educational achievement are reported in Table 2. The correlations between the MZ twin intra-pair differences in ADHD symptoms and educational achievement were significant for both boys, ranging from −0.10 to −0.32, and girls, ranging from −0.11 to −0.35. Thus, in genetically identical twin pairs, the twin with more ADHD symptoms had a lower educational achievement score than his or her co-twin.

### Between groups differences model

Table 4 lists the means and standard deviations for educational achievement and ADHD symptoms (CRS-R en CBCL), as assessed by the mother and teacher, separately for the three groups. According to mothers, inattention at age 12, was lower in the ADHD group with medication compared to the ADHD group without medication ($\beta$ = −0.83, *Cohen’s D* = −0.71, *p* < 0.001). Teachers also reported fewer inattention symptoms at age 12 in the ADHD group with medication ($\beta$ = −0.62, *Cohen’s D* = −0.50, *p* < 0.001). There were no differences in hyperactive symptoms at age 12, according to mothers ($\beta$ = 0.15, *Cohen’s D* = 0.07, *p* = 0.294) and teachers ($\beta$ = 0.54, *Cohen’s D* = 0.27, *p* = 0.024). The children in the ADHD group with medication did not differ from the children in the ADHD group without medication ($\beta$ = 0.08, *Cohen’s D* = 0.05, *p* = 0.442) with regard to ADHD symptoms at the age of 3 when, in all likelihood, none of the children were yet using methylphenidate.

**Table 4:** Means and standard deviation (SD) for ADHD symptoms and educational achievement (EA) A group without ADHD (no-ADHD group), a group with ADHD using methylphenidate (ADHD group with medication) and a group with ADHD not using methylphenidate (ADHD group without medication)

	1. No-ADHD group (N = 10,019)			2. ADHD group with medication (N = 349)			3. ADHD group without medication (N = 534)			2 versus 1			2 versus 3		
	N	Mean	SD	N	Mean	SD	N	Mean	SD	$\beta$	p	Cohen’s D	$\beta$	p	Cohen’s D
ADHD symptoms															
CPRS-R (Mother)															
Inattention	10,011	1.5	1.9	306	9.2	4.5	534	11.9	3.3	2.31	<0.001	3.80	−0.83	<0.001	−0.71
Hyperactivity	10,012	0.8	1.3	305	6.2	4.2	532	5.9	3.9	2.48	<0.001	3.67	0.15	0.294	0.07
CTRS-R (Teacher)															
Inattention	3957	1.8	2.6	101	3.7	3.0	177	5.4	3.6	0.68	<0.001	0.73	−0.62	<0.001	−0.50
Hyperactivity	3974	1.0	2.1	101	5.0	4.8	177	3.7	4.7	1.66	<0.001	1.36	0.54	0.024	0.27
CBCL (Mother)															
Age 3	8634	1.9	1.8	293	3.4	2.3	437	3.3	2.1	0.84	<0.001	0.82	0.08	0.442	0.05
Age 7	8334	2.2	2.4	250	7.2	3.9	402	5.9	3.5	1.86	<0.001	2.04	0.52	<0.001	0.36
Age 9	6927	2.1	2.3	175	7.7	3.3	323	7.2	3.7	2.09	<0.001	2.40	0.21	0.097	0.14
Age 12	9947	1.7	1.9	313	7.9	3.5	532	8.5	3.5	2.32	<0.001	3.15	−0.17	0.083	−0.17
Educational achievement															
Total score	7057	538.7	8.2	141	536.6	7.9	264	531.6	9.4	−0.23	0.008	−0.26	0.57	<0.001	0.56
Arithmetic	5156	77.9	15.7	107	75.0	15.4	162	65.2	18.4	−0.14	0.165	−0.18	0.61	<0.001	0.57
Language	5158	78.4	11.5	107	73.4	12.3	162	71.9	12.6	−0.39	<0.001	−0.43	0.15	0.258	0.12
Study skills	5151	80.0	13.1	107	76.7	13.1	161	71.2	15.5	−0.23	0.015	−0.25	0.41	0.002	0.38

*CPRS-R* short Conners’ parent rating scales, *CTRS-R* short Conners’ teacher rating scales, *CBCL* Child behavior check list

The ADHD group with medication outperformed the ADHD group without medication ($\beta$ = 0.57, *Cohen’s D* = 0.56, *p* < 0.001) on the educational achievement test at age 12 and scored almost the same as the no-ADHD group ($\beta$ = −0.23, *Cohen’s D* = −0.26, *p* = 0.008). For more than half of the children there was information on their secondary education level at ages 14 and/or 16, i.e. no-ADHD group (level 1: 32.5%; level 2: 28.1%; level 3: 39.4%), the ADHD group with medication (level 1: 63.0%; level 2: 26.0%; level 3: 11.0%) and the ADHD group without medication (level 1: 80.1%; level 2: 13.8%; level 3: 6.1%). The probability for attending the highest level of education compared to the lower levels was lower for the children in the ADHD group with medication compared to the no-ADHD group (*OR* = 0.33, *p* < 0.001), but higher compared to the ADHD group without medication (*OR* = 1.72, *p* = 0.013).

Socioeconomic status was assessed for families of children in the no-ADHD group (level 1: 2.4%; level 2: 11.6%; level 3: 40.5%; level 4: 31.7%; level 5: 13.9%), the ADHD group with medication (level 1: 7.4%; level 2: 17.2%; level 3: 41.4%; level 4: 24.2%; level 5: 9.8%) and the ADHD group without medication (level 1: 5.1%; level 2: 17.0%; level 3: 43.6%; level 4: 27.7%; level 5: 6.6%). The odds for belonging to the highest socioeconomic status level compared to the lower levels were similar for the ADHD group with medication and the ADHD group without medication (*OR* = 0.96, *p* = 0.658), but the odds were lower in the ADHD group with medication compared to the no-ADHD group (*OR* = 0.71, *p* < 0.001).

## Discussion

The aim of the present study was to determine whether the association between ADHD symptoms and lower educational achievement is best explained by a direct causal effect of ADHD symptoms on educational achievement, by genetic pleiotropy, or by confounding due to non-genetic factors. In line with earlier research a significant negative association between ADHD symptoms and educational achievement was found (Polderman et al. 2010). Children, who displayed more ADHD symptoms, as rated by their mother at the same time or 5 years earlier, scored lower on an educational achievement test. Comparing the different components of ADHD, inattentiveness and hyperactivity, suggests variation in the magnitude of the association with educational achievement. Inattentiveness is to a much greater extent related to educational achievement than hyperactivity. This is in line with a systematic review that concluded that inattention was highly predictive of educational achievement while results for hyperactivity were less conclusive (Polderman et al. 2010). One explanation might be that a relatively stable pattern of inattentive symptoms is often seen in children with ADHD compared to a decline of hyperactivity symptoms with increasing age (Biederman et al. 2000). Another explanation is that the time children spent doing their schoolwork is related to their educational achievement and having poor attention skills makes it harder to focus on classroom activities (Duncan et al. 2007). In addition, inattentive symptoms might hamper students in developing basic skills in the early grades which are often needed to learn higher level skills (Breslau et al. 2009).

Four tests were conducted to distinguish direct causality from an underlying third factor. Taken together, the tests were consistent with a direct causal effect of ADHD symptoms on educational achievement. The cross-sectional and longitudinal direct paths between ADHD symptoms and educational were significant. This was also true for the inattentive subtype of symptoms, but not for hyperactivity. Within genetically identical twin pairs, the twin who showed more ADHD symptoms scored lower on the educational achievement test than his or her co-twin. The children with ADHD, who used methylphenidate as treatment for their symptoms, scored significantly higher on an educational achievement test than the children with ADHD that did not use methylphenidate. More importantly, fewer children with ADHD using methylphenidate attended the lowest level of secondary education during adolescence compared to the group children with ADHD who did not use medication.

The effect of ADHD medication on the performance of children at school has been investigated in earlier research. A meta-analysis concluded that, when medication usage resulted in a decrease in symptoms of ADHD, children were indeed better able to stay focused, but the influence on the accuracy of the completed questions was only modest (Prasad et al. 2013). Arithmetic test scores were enhanced while the outcome was less consistent in spelling and reading. Although short-term effects have been reported, some studies looking at the effects beyond a year did not find these improvements (Parker et al. 2013). Explanations for this fading effect on educational achievement might be the development of a tolerance to the medication or the possibility that improving concentration is eventually not enough to succeed in secondary school as assignments are becoming increasingly difficult (Sharp 2014).

The children with ADHD using methylphenidate were taking this medication at age 12, when they also took the educational achievement test. However, the exact age at which the children started using methylphenidate was unknown. Information on medication usage at earlier ages was available for half of the children. At age 10, approximately 50% of the children were using methylphenidate while at age 7 only around 15% took medication. ADHD is rarely diagnosed before the age of 6 which means that probably none of the children were using methylphenidate at the age of 3.

Methylphenidate usage may be influenced by several socioeconomic, demographic and behavioral factors, implying that there could be a confounding factor that determines whether a child with ADHD is prescribed medication. Studies from North America have shown children from lower socioeconomic families are more often prescribed methylphenidate than children from higher socioeconomic status (Brownell et al. 2006; Miller et al. 2001) while a study from Israel found the opposite effect with children from higher socioeconomic status more often using methylphenidate (Jaber et al. 2014). In the Netherlands, our data did not reveal any differences when comparing the socioeconomic status of the families of children with ADHD using methylphenidate and the children not taking medication. Access to health care in the Netherlands is relatively equal for all people. This means that socioeconomic status is not underlying the higher educational achievement in the group of children with ADHD that take methylphenidate.

The finding that ADHD symptoms and educational achievement are negatively associated while controlling for confounding by genetic variation and family environment, is an important first step in establishing a direct causal effect of ADHD on low educational achievement. What must be noted is that the direct causal hypothesis can only be proven using experimental data. However, the method of estimating the direct path between ADHD symptoms and educational achievement while using a Cholesky decomposition to filter out the unmeasured confounds is a good approximation. As family environment and genotype (partly) is shared by the twins this cannot be a potential explanation for differences between children of a twin pair. A promising other new method that might be able to answer the question what the ‘true’ direct causal effect is of ADHD on educational achievement is Mendelian randomization combined with bivariate twin modeling (Pierce et al. 2011; Smith and Hemani 2014). Using this method the direct causal effect of ADHD on educational achievement can also be corrected for genetic pleiotropic effects and for the effects of environmental factors that are related to both ADHD and educational achievement.

A drawback is that it was not possible to distinguish more complex mechanisms, such as bidirectional causality. Bidirectional causality implies that ADHD symptoms lead to lower educational achievement and in turn these problems at school may enhance the already existing symptoms. Various direction of causality models have been proposed to study bidirectional mechanisms (Heath et al. 1993; Duffy and Martin 1994). However, to resolve the direction of the causal association these models require a substantial difference in heritability between the two phenotypes under study, which is not the case for ADHD symptoms and educational achievement.

To conclude, a direct causal effect seems to, at least partly, underlie the association between ADHD and low educational achievement. A practical implication is that, when the prescription of medication, e.g. methylphenidate, leads to a reduction in ADHD symptoms, it can also, as a positive side effect, have an enhancing influence on educational achievement. There is also some evidence that psychological interventions, e.g. behavioral therapy, parent training and social skills training, have beneficial effects on ADHD symptoms in school-ages children (Serrano-Troncoso et al. 2013), in which case an improvement in educational achievement is also expected. One must keep in mind that the effect on educational achievement may be larger for children displaying inattentive symptoms compared to children mainly demonstrating hyperactive symptoms.

## Electronic supplementary material

Below is the link to the electronic supplementary material.

Supplementary material 1 (DOCX 29 KB)
