# Supplementary material for: Attention Deficit Hyperactivity Disorder Symptoms and Low Educational Achievement: Evidence Supporting A Causal Hypothesis
Source: Behav Genet. 2017 Feb 13;47(3):278–89. doi: 10.1007/s10519-017-9836-4 (PMC5403868; doi:10.1007/s10519-017-9836-4)
Supplement: Supplementary file 1 — Supplementary material 1 (DOCX 29 KB) [file 10519_2017_9836_MOESM1_ESM.docx]

**Table S1 *Model fitting*** Univariate genetic model results for ADHD symptoms (at age 12) and educational achievement (at age 12)

|  | **ep** | **-2ll** | **df** | **model** | **χ2** | **Δdf** | **p** |
| --- | --- | --- | --- | --- | --- | --- | --- |
| **Inattention** |  |  |  |  |  |  |  |
| 0 Saturated | 25 | 38305.3 | 13137 | - | - | - | - |
| 1 Birth Order - Equal Means | 21 | 38307.7 | 13141 | 0 | 2.42 | 4 | .659 |
| 2 Birth Order - Equal Variances | 17 | 38311.1 | 13145 | 1 | 3.39 | 4 | .494 |
| 3 Zygosity - Equal Means | 13 | 38339.5 | 13149 | 2 | 28.35 | 4 | <.001 |
| 4 Zygosity - Equal Variances | 13 | 38321.2 | 13149 | 2 | 10.09 | 4 | .039 |
| 5 Sex - Equal Means | 10 | 38789.8 | 13152 | 4 | 468.59 | 3 | <.001 |
| 6 Sex - Equal Variances | 12 | 38395.0 | 13150 | 4 | 73.78 | 1 | <.001 |
| 7 ACE | 8 | 38471.5 | 13154 | 0 | 166.20 | 17 | <.001 |
| 8 ADE | 8 | 38351.6 | 13154 | 0 | 46.36 | 17 | <.001 |
| 9 ADE - No Sex Differences | 6 | 38375.5 | 13156 | 8 | 23.81 | 2 | <.001 |
| 10 AE - Girls | 7 | 38371.2 | 13155 | 8 | 19.55 | 1 | <.001 |
| 11 AE - Boys | 7 | 38381.9 | 13155 | 8 | 30.24 | 1 | <.001 |
| **Hyperactivity** |  |  |  |  |  |  |  |
| 0 Saturated | 25 | 32349.5 | 13146 | - | - | - | - |
| 1 Birth Order - Equal Means | 21 | 32355.8 | 13150 | 0 | 6.37 | 4 | .173 |
| 2 Birth Order - Equal Variances | 17 | 32356.6 | 13154 | 1 | .73 | 4 | .947 |
| 3 Zygosity - Equal Means | 13 | 32393.8 | 13158 | 2 | 37.20 | 4 | <.001 |
| 4 Zygosity - Equal Variances | 13 | 32386.7 | 13158 | 2 | 30.11 | 4 | <.001 |
| 5 Sex - Equal Means | 14 | 32914.2 | 13157 | 2 | 557.63 | 3 | <.001 |
| 6 Sex - Equal Variances | 14 | 32609.9 | 13157 | 2 | 253.30 | 3 | <.001 |
| 7 ACE | 8 | 32466.0 | 13163 | 0 | 116.54 | 17 | <.001 |
| 8 ADE | 8 | 32426.0 | 13163 | 0 | 76.56 | 17 | <.001 |
| 9 ADE - No Sex Differences | 6 | 32434.8 | 13165 | 8 | 8.75 | 2 | .013 |
| 10 AE | 5 | 32525.7 | 13166 | 9 | 90.95 | 1 | <.001 |
| **ADHD** |  |  |  |  |  |  |  |
| 0 Saturated | 25 | 42867.4 | 13141 | - | - | - | - |
| 1 Birth Order - Equal Means | 21 | 42872.3 | 13145 | 0 | 4.95 | 4 | .292 |
| 2 Birth Order - Equal Variances | 17 | 42877.7 | 13149 | 1 | 5.37 | 4 | .251 |
| 3 Zygosity - Equal Means | 13 | 42914.9 | 13153 | 2 | 37.20 | 4 | <.001 |
| 4 Zygosity - Equal Variances | 13 | 42881.7 | 13153 | 2 | 3.97 | 4 | .410 |
| 5 Sex - Equal Means | 10 | 43462.7 | 13156 | 4 | 581.05 | 3 | <.001 |
| 6 Sex - Equal Variances | 12 | 42919.6 | 13154 | 4 | 37.99 | 1 | <.001 |
| 7 ACE | 8 | 42973.3 | 13158 | 0 | 105.90 | 17 | <.001 |
| 8 ADE | 8 | 42919.9 | 13158 | 0 | 52.55 | 17 | <.001 |
| 9 ADE - No Sex Differences | 6 | 42948.7 | 13160 | 8 | 28.74 | 2 | <.001 |
| 10 AE - Girls | 7 | 42924.0 | 13159 | 8 | 4.09 | 1 | .043 |
| 11 AE - Boys | 6 | 42977.7 | 13160 | 10 | 53.72 | 1 | <.001 |
| **Educational Achievement** |  |  |  |  |  |  |  |
| 0 Saturated | 25 | 71164.0 | 10318 | - | - | - | - |
| 1 Birth Order - Equal Means | 21 | 71173.9 | 10322 | 0 | 9.89 | 4 | .042 |
| 2 Birth Order - Equal Variances | 17 | 71177.0 | 10326 | 1 | 3.11 | 4 | .540 |
| 3 Zygosity - Equal Means | 13 | 71186.0 | 10330 | 2 | 9.05 | 4 | .060 |
| 4 Zygosity - Equal Variances | 9 | 71191.4 | 10334 | 3 | 5.34 | 4 | .254 |
| 5 Sex - Equal Means | 8 | 71241.0 | 10335 | 4 | 49.66 | 1 | <.001 |
| 6 Sex - Equal Variances | 8 | 71197.6 | 10335 | 4 | 6.26 | 1 | .012 |
| 7 ACE | 8 | 71191.4 | 10335 | 0 | 27.42 | 17 | .052 |
| 8 ADE | 8 | 71196.4 | 10335 | 0 | 32.45 | 17 | .013 |
| 9 ACE - No Sex Differences | 6 | 71192.2 | 10337 | 7 | .76 | 2 | .684 |
| 10 AE | 5 | 71198.6 | 10338 | 9 | 6.45 | 1 | .011 |

^ep = estimated parameters; -2ll = minus 2 loglikelihood; χ2 = chi square; df = degrees of freedom^

**Table S2 *Model fitting*** Univariate genetic model results for ADHD symptoms (at age 7)

|  | **ep** | **-2ll** | **df** | **model** | **χ2** | **Δdf** | **p** |
| --- | --- | --- | --- | --- | --- | --- | --- |
| **Inattention** |  |  |  |  |  |  |  |
| 0 Saturated | 25 | 38403.8 | 12979 | - | - | - | - |
| 1 Birth Order - Equal Means | 21 | 38429.5 | 12983 | 0 | 25.71 | 4 | <.001 |
| 2 Birth Order - Equal Variances | 17 | 38433.2 | 12987 | 1 | 3.67 | 4 | .453 |
| 3 Zygosity - Equal Means | 13 | 38436.7 | 12991 | 2 | 3.53 | 4 | .473 |
| 4 Zygosity - Equal Variances | 9 | 38444.6 | 12995 | 3 | 7.89 | 4 | .096 |
| 5 Sex - Equal Means | 8 | 38762.4 | 12996 | 4 | 317.74 | 1 | <.001 |
| 6 Sex - Equal Variances | 8 | 38498.2 | 12996 | 4 | 53.57 | 1 | <.001 |
| 7 ACE | 8 | 38495.2 | 12996 | 0 | 91.41 | 17 | <.001 |
| 8 ADE | 8 | 38444.8 | 12996 | 0 | 40.96 | 17 | <.001 |
| 9 ADE - No Sex Differences | 6 | 38449.4 | 12998 | 8 | 4.60 | 2 | .100 |
| 10 AE | 5 | 38518.4 | 12999 | 9 | 69.04 | 1 | <.001 |
| **Hyperactivity** |  |  |  |  |  |  |  |
| 0 Saturated | 25 | 34770.1 | 13066 | - | - | - | - |
| 1 Birth Order - Equal Means | 21 | 34779.5 | 13070 | 0 | 9.41 | 4 | .052 |
| 2 Birth Order - Equal Variances | 17 | 34783.4 | 13074 | 1 | 3.91 | 4 | .419 |
| 3 Zygosity - Equal Means | 13 | 34796.3 | 13078 | 2 | 12.91 | 4 | .012 |
| 4 Zygosity - Equal Variances | 9 | 34802.2 | 13082 | 3 | 5.86 | 4 | .210 |
| 5 Sex - Equal Means | 8 | 35392.9 | 13083 | 4 | 590.75 | 1 | <.001 |
| 6 Sex - Equal Variances | 8 | 34882.9 | 13083 | 4 | 80.79 | 1 | <.001 |
| 7 ACE | 8 | 34804.0 | 13083 | 0 | 33.92 | 17 | .009 |
| 8 ADE | 8 | 34802.7 | 13083 | 0 | 32.6 | 17 | .013 |
| 9 ADE - No Sex Differences | 6 | 3816.43 | 13085 | 8 | 13.72 | 2 | .001 |
| 10 AE - Girls | 7 | 34802.7 | 13084 | 8 | .01 | 1 | .935 |
| 11 AE - Boys | 6 | 34806.0 | 13085 | 10 | 3.24 | 1 | .072 |
| **ADHD** |  |  |  |  |  |  |  |
| 0 Saturated | 25 | 43101.0 | 13047 | - | - | - | - |
| 1 Birth Order - Equal Means | 21 | 43127.1 | 13051 | 0 | 26.14 | 4 | <.001 |
| 2 Birth Order - Equal Variances | 17 | 43128.6 | 13055 | 1 | 1.50 | 4 | .827 |
| 3 Zygosity - Equal Means | 13 | 43139.3 | 13059 | 2 | 10.63 | 4 | .031 |
| 4 Zygosity - Equal Variances | 9 | 43148.6 | 13063 | 3 | 9.34 | 4 | .053 |
| 5 Sex - Equal Means | 8 | 43555.4 | 13064 | 4 | 406.80 | 1 | <.001 |
| 6 Sex - Equal Variances | 8 | 43179.8 | 13064 | 4 | 31.23 | 1 | <.001 |
| 7 ACE | 8 | 43190.2 | 13064 | 0 | 89.20 | 17 | <.001 |
| 8 ADE | 8 | 43149.0 | 13064 | 0 | 48.04 | 17 | <.001 |
| 9 ADE - No Sex Differences | 6 | 43157.1 | 13066 | 8 | 8.09 | 2 | .017 |
| 10 AE | 5 | 43210.7 | 13067 | 9 | 53.53 | 1 | <.001 |

^ep = estimated parameters; -2ll = minus 2 loglikelihood; χ2 = chi square; df = degrees of freedom^

**Table S3 *Cholesky Decomposition*** Standardized estimates of the percentages of variance in ADHD symptoms and educational achievement attributed to additive genetic (A), dominant genetic (D), common environmental (C) and unique environmental (E) effects

|  | **Boys** | | | | **Girls** | | | |
| --- | --- | --- | --- | --- | --- | --- | --- | --- |
|  | **A** | **D** | **C** | **E** | **A** | **D** | **C** | **E** |
| **Age 7** |  |  |  |  |  |  |  |  |
| Inattention | 41% | 34% | - | 24% | 30% | 43% | - | 27% |
| Hyperactivity | 72% | 11% | - | 17% | 80% | 0% | - | 20% |
| ADHD | 60% | 19% | - | 22% | 32% | 44% | - | 24% |
| **Age 12** |  |  |  |  |  |  |  |  |
| Inattention | 5% | 72% | - | 22% | 23% | 48% | - | 30% |
| Hyperactivity | 38% | 41% | - | 22% | 51% | 25% | - | 24% |
| ADHD | 31% | 51% | - | 18% | 55% | 21% | - | 24% |
| Educational Achievement | 71% | - | 10% | 18% | 79% | - | 4% | 18% |
